# Atracurium-Induced Bronchospasm With Flat Capnograph at Induction of General Anaesthesia: A Case Report

**DOI:** 10.7759/cureus.54251

**Published:** 2024-02-15

**Authors:** Alfie Wright, Thomas Leahy

**Affiliations:** 1 Anaesthetics, Southend University Hospital, Southend, GBR

**Keywords:** case report, anesthetic planning, induction, vl, video laryngoscopy, premedication, atracurium, bronchospasm, general anesthesia

## Abstract

Benzylisoquinolinium neuromuscular blocking agents can precipitate bronchospasm either through allergy/anaphylaxis or isolated stimulation of mast cell histamine release. This report presents a 75-year-old female who attended the day surgery unit for a rigid cystoscopy under general anaesthesia. She had a hyper-reactive airway history of mild historic asthma and sensitivity to aerosols. After administration of atracurium at induction of anaesthesia, ventilation became challenging with no chest rise and a flat CO_2_ trace. Repeat video laryngoscopy confirmed correct endotracheal tube position. The patient remained cardiovascularly stable with no mucocutaneous signs of anaphylaxis. Administration of high flow oxygen, sevoflurane, salbutamol and magnesium sulfate led to gradual improvement and normalisation of respiratory parameters. Surgery was postponed. This report highlights atracurium as an important trigger of bronchospasm at induction of anaesthesia, and illustrates that in rare cases a flat capnograph does not always indicate a mispositioned airway device. Several aspects of the anaesthetic plan for this patient were suboptimal given her respiratory history, namely, the choice of mode of anaesthesia and choice of neuromuscular blocking agent. These factors are discussed in the context of anaesthetic planning for patients presenting with features suggesting high bronchospastic risk.

## Introduction

Bronchospasm during anaesthesia is the result of exacerbated underlying airway hyper-reactivity. Through impedance of ventilation, it can be fatal if not promptly recognised and treated. Perioperative bronchospasm can be recognised by high airway pressures, desaturation, and changes in the CO_2_ waveform [1]. Rarely, severe cases may present with a flat CO_2_ trace or silent chest [2,3]. It is managed by giving high-flow oxygen, increasing the depth of anaesthesia, administration of antispasmodic pharmacological therapies, and employing ventilatory techniques to mitigate breath-stacking [1,4].

Although bronchospasm during anaesthesia can be a manifestation of anaphylaxis, most episodes are not attributable to allergy: a retrospective study found only 22 of 103 cases of bronchospasm to be allergic/anaphylactic in nature [3]. Other risk factors and triggers implicated in the aetiology of bronchospasm include poorly-controlled asthma, respiratory infection, various medications, smoking, inadequate depth of anaesthesia, and mechanical stimulation of the airway [5-8]. However, in many cases there are no identifiable risk factors [3,7].

Benzylisoquinolinium neuromuscular blocking agents (NMBAs) such as atracurium are among the medications which can trigger bronchospasm, via a mechanism involving mast cell histamine release [6,8]. The reported incidence of bronchospasm after atracurium varies between 0.2% and 1.5% [9,10].

This report describes an occurrence of severe bronchospasm at induction of general anaesthesia, immediately following administration of atracurium, presenting with a silent chest and flat CO_2_ trace. The case highlights that atracurium remains an important trigger of bronchospasm at induction of anaesthesia, and illustrates that in rare cases, a flat capnograph does not indicate a mispositioned airway device. Lastly, strategies for reducing bronchospasm risk in susceptible patients are discussed.

## Case presentation

A 75-year-old female was scheduled for a day-case rigid cystoscopy to investigate MRI-demonstrated bladder wall thickening in the context of persistent culture-negative lower urinary tract symptoms. General anaesthesia (GA) was chosen as the anaesthetic method, taking into account the patient’s and surgeon’s preferences. The patient had a past medical history of relapsed malignant melanoma with metastatic lung nodules, type 2 diabetes mellitus, arachnoiditis, osteoarthritis, established peripheral neuropathy secondary to immunotherapy, and essential hypertension. Her body mass index (BMI) was 37 kg/m^2^. Her regular medications comprised: nivolumab, amitriptyline, gabapentin, indapamide, lercanidipine, metformin and omeprazole. She was also prescribed an “as-required” salbutamol inhaler for an historic diagnosis of mild asthma; however, she had not required this medication for several years. She reported a sensitivity to some aerosol air fresheners, with exposure causing mild dyspnoea. There was no other history of allergy or sensitivity. She denied any recent respiratory symptoms, however whether or not she had been recently exposed to aerosols was not fully interrogated and there were no up-to-date spirometry data to support an adequate level of asthmatic control. 

Her prior surgical history included abdominal hysterectomy, bilateral total knee replacements, and cholecystectomy; and there were no anaesthetic complications during any of these procedures. Her exercise tolerance was moderate, being able to ascend stairs comfortably without dyspnoea, and she was a lifelong non-smoker. She did not drink alcohol and there was no recent travel history.

On assessment, she was Mallampati grade 2 [11] and displayed adequate mouth opening and range of neck movement. Preoperative blood tests were normal. Her most recent electrocardiogram (ECG) showed sinus rhythm with poor R-wave progression and no other abnormalities.

Upon arrival in the anaesthetic room, monitoring was placed on the patient in accordance with the Association of Anaesthetists of Great Britain and Ireland (AAGBI) recommendations [12]. Intravenous (IV) access was obtained, and the patient was pre-oxygenated. General anaesthesia (GA) was induced with IV injection of 200mcg of fentanyl and 150mg of propofol. Sevoflurane was commenced and initial bag/mask ventilation was easy with a good capnography trace. An absent eyelash reflex was demonstrated, and an appropriate age-corrected Minimum Alveolar Concentration (MAC) of sevoflurane was reached. To facilitate endotracheal intubation, 35mg of atracurium was administered intravenously. Approximately two minutes after the atracurium injection, ventilation became progressively challenging, with increasing airway pressures and worsening chest movement. Notably, the CO_2_ trace gradually fell in amplitude until it flattened altogether. Initial airway maneuvers (oropharyngeal airway insertion, jaw thrust provided by a second anaesthetist) were unsuccessful, as was an attempt to ventilate with an i-gel^TM^ supraglottic airway. A first attempt at endotracheal intubation was made with direct laryngoscopy (DL), which provided a modified Cormack-Lehane grade 2b view [13], a size 7 endotracheal tube (ETT) was railroaded over a gum-elastic bougie. Adequate ventilation was not possible, and so reintubation was performed using video laryngoscopy (VL) and a bougie. The ETT was seen to pass between the vocal cords; however, ventilation remained unachievable with a flat capnograph. VL was again performed to check the position of the ETT, and a consensus among the team was reached that, notwithstanding the lack of CO_2_ trace, the ETT was correctly sited passing through the glottis.

Progressive desaturation was observed during intubation, and the SpO_2_ was ~50% after the final confirmation of ETT position. The emergency alarm was activated, 100% high flow oxygen was administered with maximal sevoflurane, and a 50 mg bolus of propofol was given IV. Manual ventilation via the ETT was attempted; however, the chest was silent on auscultation, and there was no clinical response or restoration of a CO_2_ trace. Throughout, the patient remained normotensive, sinus tachycardic, and peripherally well-perfused. There was no rash or mucosal swelling found on examination.

**Figure 1:**
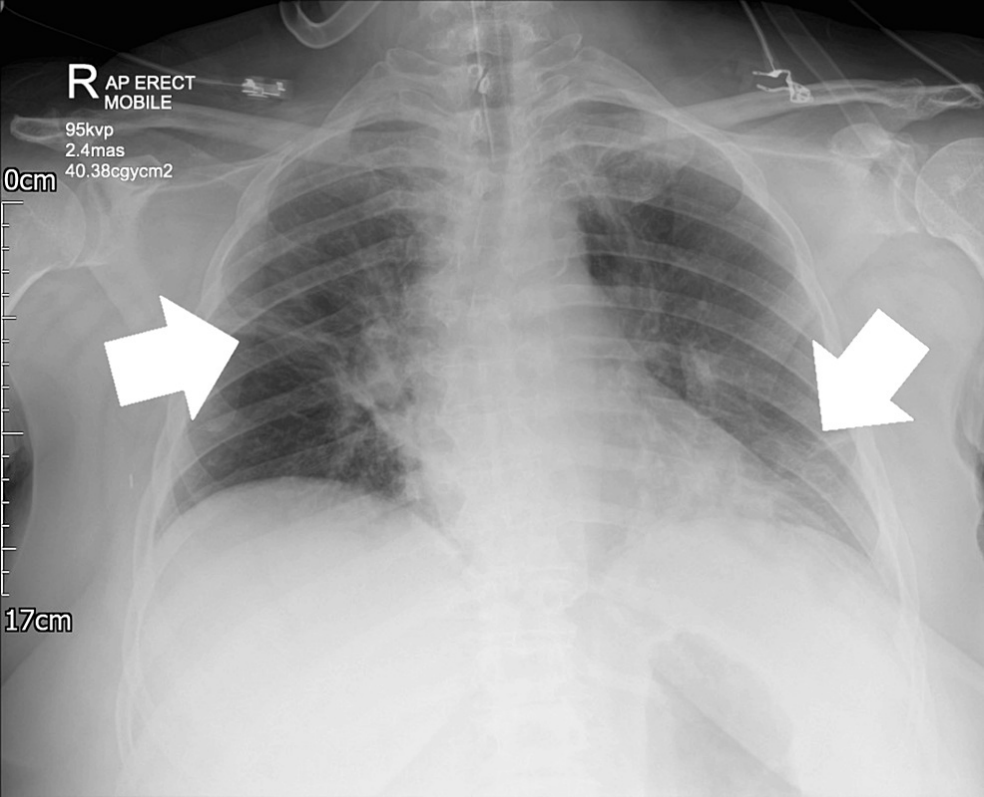
The patient’s plain film chest radiograph taken in the anaesthetic room The report obtained from the duty consultant radiologist is as follows: An endotracheal tube is in situ and appears adequately positioned with the tip approximately 5 cm above the carina. Linear atelectasis at the right mid zone and left lower zone with patchy opacity consistent with infection and/or aspiration. No pneumothorax. Normal heart size. No bony abnormality.

Five milligrams of salbutamol were administered through the ETT via a nebuliser and 2g of magnesium sulfate was given slowly IV. A gradual improvement in SpO_2 _and airway pressures was noted with the re-establishment of a CO_2_ trace, now seen with a “shark fin” appearance suggestive of airway obstruction [14]. Quiet breathing sounds were heard on auscultation, without wheeze. A chest X-ray (CXR) in the anaesthetic room appeared grossly normal and ruled out pneumothorax or lobar collapse, though the formal report suggested linear atelectasis at the right mid zone and left lower zone (see Figure 1). After ~45 minutes, respiratory parameters had normalised. It was agreed with the surgical team that her procedure be deferred to allow for prolonged respiratory monitoring unaffected by surgical stimulus, and the patient was subsequently extubated and transferred to recovery. Blood tests taken during the event and repeated two hours later confirmed a normal full blood count (FBC), electrolytes, CRP, thyroid function tests (TFTs), and mast cell tryptase levels.

Following review by intensivist physicians, the patient was monitored for the rest of the day and discharged home that evening. She has had no further respiratory compromise. Given the elective nature of her surgery, it was agreed that her rescheduled operation be postponed until after several investigations are completed. These included formal allergy testing to rule out an allergy to atracurium, spirometry with reversibility to assess her level of asthmatic control, and a repeat CXR.

## Discussion

Bronchospasm with flat capnography: implications for confirmation of ETT position 

Several features of the present case indicate bronchospasm: increased airway pressures, desaturation, CO_2_ trace changes, and reduced air entry on auscultation of the chest [3,14]. Interestingly, a flat CO_2_ trace was seen, which was reported only once in a review of 103 cases of perioperative bronchospasm [3], and far more commonly signifies a misplaced ETT such as in oesophageal intubation [15]. The AAGBI’s Quick Reference Handbook features the exclusion of a mispositioned airway device in the algorithm for the management of bronchospasm [4]. This is usually performed using capnography in the context of the Royal College of Anaesthetists’ “no trace, wrong place” initiative [16]. Therefore, the possibility of a flat CO_2_ trace during severe bronchospasm is an important consideration.

Consensus guidelines have been produced by the Project for Universal Management of Airways and international airway societies, which aim to prevent oesophageal intubation [15]. The authors address bronchospasm as an anecdotal cause of absent sustained expired CO_2_ and warn that this supposition has previously contributed to patient morbidity and mortality due to unrecognised oesophageal intubation [15,17,18]. Consequently, if bronchospasm is suspected, the authors recommend ventilation with adequate ventilatory pressure and expiratory time in the first instance. These techniques reduce breath-stacking and gas-trapping [1], and frequently restore a CO_2_ trace [15]. If this is unsuccessful, then alternative techniques should be employed to exclude oesophageal intubation: repeat laryngoscopy (VL whenever feasible), ideally followed by one supplementary technique such as flexible bronchoscopy or ultrasonography [15]. In both our patient and in another recently reported incidence of severe bronchospasm [2], repeat VL was utilised to demonstrate clearly that the ETT was passing between the glottis, enabling a timely consensus agreement amongst the anaesthetic team that oesophageal intubation was not responsible for the absence of sustained expired CO_2_.

The aetiology of bronchospasm in our patient

In an analysis of the aetiology of bronchospasm in the present case, there are several precipitous and background factors that must be evaluated. Mechanical stimulation of the airway has been described as an important trigger of bronchospasm [7] but seems an unlikely culprit in our patient given that bronchospasm occurred prior to intubation following a period of easy mask ventilation. Inadequate depth of anaesthesia also warrants consideration [8], but it also seems improbable to have played a significant role here as the eyelash reflex was abolished and an appropriate MAC of sevoflurane was reached. 

Rather, in view of the chronological correlation of atracurium administration and clinical deterioration, atracurium appears a plausible precipitous agent. This is supported by the time course of the reaction in our patient, which is consistent with data demonstrating a median time of two minutes between administration of atracurium and adverse reaction [10]. An anaphylactic reaction should be considered, but seems unlikely due to various aspects: absence of mucocutaneous involvement, normotension, duration of event <60 minutes, and two normal-range mast cell tryptase assays [19]. Atracurium-mediated mast cell histamine release appears a more likely mode of causality [6,8] and has been widely reported with an incidence between 0.2% and 1.5% [9,10].

Several features of our patient’s medical history suggest a level of airway hyper-reactivity, which might have contributed to bronchospasm. Firstly, a historical diagnosis of mild asthma: while our patient denied any recent respiratory symptoms and had not needed to use her salbutamol inhaler in several years, the absence of up-to-date spirometry data renders it possible that her asthma was not adequately controlled. Furthermore, it was not documented whether the patient had been recently exposed to any aerosols to which she reported respiratory sensitivity. Proper interrogation of this reported sensitivity and arrangement of spirometry with reversibility testing might represent areas where the preoperative assessment of this patient could have been improved upon [8]. It is worth noting also that obesity is becoming increasingly recognised as a risk factor for bronchospasm. For example, a prospective observational study compared patients undergoing elective laparoscopic surgery and reported that the incidence of bronchospasm was significantly higher in patients with a BMI >35 kg/m^2^ [20]. Elsewhere, logistic regression modelling has demonstrated an association between BMI and the onset of airway hyper-responsiveness [21]. Our patient’s BMI was 37 kg/m^2^, therefore perhaps further suggesting background airway hyper-reactivity. 

Overall, our patient had asthma controlled to an uncertain degree, aerosol sensitivity, and an elevated BMI. Upon this multifaceted background of airway hyper-reactivity, atracurium seems to have been the bronchospastic precipitant.

Reducing the risk of bronchospasm: anaesthetic planning for high-risk patients

A key learning point from this case relates to anaesthetic planning for patients who have either demonstrated bronchospasm under general anaesthesia, or whose preoperative assessment elucidates features suggesting high bronchospastic risk (for instance: smokers, asthmatics, and concurrent respiratory infection) [5,7].

In either case, opting for regional anaesthesia (RA) where possible rather than GA seems prudent as this altogether avoids direct airway instrumentation and the requirement for NMBAs. Though note, it is suggested that anxiety and pain during RA can themselves precipitate bronchospasm [8]. The choice of anaesthetic should take into account a thorough clinical assessment, the planned surgery, and the preferences of all involved. In the present case, GA was chosen after taking into account the preferences of the patient and surgeon, as well as the anticipated brevity of the procedure itself. However, with a background of airway hyper-reactivity, RA would have likely been a safer approach that should have been offered to our patient alongside proper counselling as to the risk of bronchospasm with GA. Needless to say, RA will be highly preferential when our patient presents for her elective cystoscopy given her profound bronchospasm at induction of GA.

Another improvable area of the anaesthetic plan in the present case is the choice of NMBA. With a background suggestive of airway hyper-reactivity, atracurium is unlikely to represent the safest paralytic agent given the risk of bronchospasm [6]. Should a GA be necessary for a high-risk patient such as ours, and if paralysis is required, an alternative NMBA should be considered. For example, the aminosteroids rocuronium and vecuronium do not cause histamine release and might, therefore, be more appropriate agents [22,23]. Unfortunately, research is scarce as to whether bronchospastic events with one NMBA can sensitise a patient to another NMBA. While the benzylisoquinolinium and aminosteroid NMBAs have distinct chemical structures, which would intuitively seem to render such interclass cross-sensitisation less likely, further research is required to investigate this possibility.

Premedication is another useful strategy to be considered in high-risk patients. Administration of IV antihistamines [24] or magnesium prior to induction of anaesthesia has been suggested to reduce the chance of bronchospasm, as have inhaled beta-2 agonists and corticosteroids [1]. Premedication was not carried out in the present case, representing another part of the anaesthetic plan which can be optimised when the patient next attends surgery. Indeed, in a similar report of bronchospasm at induction, premedication was successful at preventing bronchospasm during a subsequent surgery [2], although here atracurium was not implicated. Another interesting finding is that slow injection of NMBAs (over 75 seconds versus rapid injection) has been associated with a reduced incidence of adverse reactions [24], a fairly simple strategy that could be incorporated into future anaesthetic planning for our patients.

Irrespective of the anaesthetic plan, judicious optimisation of patients prior to elective surgery remains paramount in reducing the risk of bronchospasm [8]. If respiratory sensitivities are reported, such as to aerosols, as was the case for our patient, these should be fully interrogated and the possibility of recent exposure should be assessed. Anaesthetists should ensure proper control of asthma, arranging spirometry with reversibility testing if this is uncertain. Smoking cessation should be encouraged: longer periods of smoking cessation prior to elective surgery are associated with fewer perioperative complications [5]. One should also consider postponing surgery if there is a suggestion of respiratory infection, particularly in children [7,8].

## Conclusions

Atracurium-mediated histamine release can trigger bronchospasm during general anaesthesia. In rare instances, severe bronchospasm can present with a flat CO_2_ trace. If bronchospasm is suspected as the cause of a flat capnograph, then ventilation with adequate ventilatory pressure and expiratory time should be ensured. If this is unsuccessful at restoring sustained expired CO_2_, then alternative techniques are required to rule out oesophageal intubation; ideally, repeat video laryngoscopy plus one supplementary technique such as flexible bronchoscopy.

Proper assessment of patient factors indicating background airway hyper-reactivity can assist in identifying patients at a higher risk of bronchospasm. In high-risk patients, regional anaesthesia should be considered as this avoids mechanical stimulation of the airway (a common bronchospastic trigger). If general anaesthesia is chosen for a high-risk patient, atracurium should be avoided in favour of aminosteroid neuromuscular blocking agents, which do not cause histamine release. Respiratory issues such as asthma should be optimised. Premedication with antihistamines, magnesium, inhaled beta-2 agonists, or corticosteroids can reduce the chance of bronchospasm in high-risk patients.
